# Interindividual Variation in Dietary Carbohydrate Metabolism by Gut Bacteria Revealed with Droplet Microfluidic Culture

**DOI:** 10.1128/mSystems.00864-19

**Published:** 2020-06-30

**Authors:** Max M. Villa, Rachael J. Bloom, Justin D. Silverman, Heather K. Durand, Sharon Jiang, Anchi Wu, Eric P. Dallow, Shuqiang Huang, Lingchong You, Lawrence A. David

**Affiliations:** a Department of Molecular Genetics and Microbiology, Duke University, Durham, North Carolina, USA; b Center for Genomic and Computational Biology, Duke University, Durham, North Carolina, USA; c University Program in Genetics and Genomics, Duke University, Durham, North Carolina, USA; d Program in Computational Biology and Bioinformatics, Duke University, Durham, North Carolina, USA; e College of Information Science and Technology, Penn State University, University Park, Pennsylvania, USA; f Department of Biomedical Engineering, Duke University, Durham, North Carolina, USA; g Shenzhen Institute of Synthetic Biology, Shenzhen Institutes of Advanced Technology, Chinese Academy of Sciences, Shenzhen, People’s Republic of China; h Department of Medicine, Penn State University, Hershey, Pennsylvania, USA; University of Pennsylvania

**Keywords:** microbiome, microfluidics, bacteria, gut, prebiotics, polysaccharides, diet, droplet, fiber

## Abstract

Culture and screening of gut bacteria enable testing of microbial function and therapeutic potential. However, the diversity of human gut microbial communities (microbiota) impedes comprehensive experimental studies of individual bacterial taxa. Here, we combine advances in droplet microfluidics and high-throughput DNA sequencing to develop a platform for separating and assaying growth of microbiota members in picoliter droplets (MicDrop). MicDrop enabled us to cultivate 2.8 times more bacterial taxa than typical batch culture methods. We then used MicDrop to test whether individuals possess similar abundances of carbohydrate-degrading gut bacteria, using an approach which had previously not been possible due to throughput limitations of traditional bacterial culture techniques. Single MicDrop experiments allowed us to characterize carbohydrate utilization among dozens of gut bacterial taxa from distinct human stool samples. Our aggregate data across nine healthy stool donors revealed that all of the individuals harbored gut bacterial species capable of degrading common dietary polysaccharides. However, the levels of richness and abundance of polysaccharide-degrading species relative to monosaccharide-consuming taxa differed by up to 2.6-fold and 24.7-fold, respectively. Additionally, our unique dataset suggested that gut bacterial taxa may be broadly categorized by whether they can grow on single or multiple polysaccharides, and we found that this lifestyle trait is correlated with how broadly bacterial taxa can be found across individuals. This demonstration shows that it is feasible to measure the function of hundreds of bacterial taxa across multiple fecal samples from different people, which should in turn enable future efforts to design microbiota-directed therapies and yield new insights into microbiota ecology and evolution.

**IMPORTANCE** Bacterial culture and assay are components of basic microbiological research, drug development, and diagnostic screening. However, community diversity can make it challenging to comprehensively perform experiments involving individual microbiota members. Here, we present a new microfluidic culture platform that makes it feasible to measure the growth and function of microbiota constituents in a single set of experiments. As a proof of concept, we demonstrate how the platform can be used to measure how hundreds of gut bacterial taxa drawn from different people metabolize dietary carbohydrates. Going forward, we expect this microfluidic technique to be adaptable to a range of other microbial assay needs.

## Abstract (precis)

Bacterial culture and assay are components of basic microbiological research, drug development, and diagnostic screening. However, community diversity can make it challenging to comprehensively perform experiments involving individual microbiota members. Here, we present a new microfluidic culture platform that makes it feasible to measure the growth and function of microbiota constituents in a single set of experiments. As a proof of concept, we demonstrate how the platform can be used to measure how hundreds of gut bacterial taxa drawn from different people metabolize dietary carbohydrates. Going forward, we expect this microfluidic technique to be adaptable to a range of other microbial assay needs.

## INTRODUCTION

Culture-based assays can reveal important functional differences between individuals’ gut microbial communities. Such differences are often not evident from 16S rRNA sequencing studies that do not resolve bacterial taxonomy below the species level (1). Culture-based studies are capable of resolving strain-level functional variation, which can drive interindividual variation in microbiome-associated health and disease outcomes. Selective growth assays, for example, have long been used to identify individuals harboring pathogenic strains of commensal gut microbial taxa (2). Inhibition assays have also been used to reveal strain variation within bacterial species that determines whether individuals’ microbiota can resist pathogens (3), and carbon utilization screens have shown that strains of the same bacterial species isolated from different people can differ in their capacity to metabolize dietary carbohydrates (4–6).

Still, a key challenge for culture-based studies in human gut microbiota research has been the need to increase the throughput of bacterial assays. It has been calculated that millions of microbial colonies would need to be cultured in order to sample the diversity now typically captured in metagenomic analyses of human gut microbiota (7). Such calculations reflect the diversity of human gut microbiota; culture-independent methods based on high-throughput sequencing of the 16S rRNA gene have shown that the average individual harbors hundreds of distinct enteric bacterial strains (8–11). Moreover, unrelated individuals do not share bacterial strains in common (12), and, because most gut taxa are rare, exhaustive capture of bacterial species from even a single stool sample requires extensive colony picking (13, 14). To reduce the human effort needed for such experiments, state-of-the-art culture assays leverage plate-handling and liquid-handling robots. However, even these automated systems are limited by the same physical constraints as typical plate-based culture methods, which grow bacteria in wells ranging from centimeters to millimeters in diameter. Even relying on 96-well and 384-well plates, conventional large-scale culture efforts may require automation to load and handle dozens of plates within an anaerobic chamber (15), ultimately limiting throughput to the study of tens of strains (15, 16).

An alternative approach is to culture bacteria in lower volumes (nanoliters to picoliters). Devices comprised of thousands of microscale compartments have been used to culture laboratory strains of both bacteria and fungi (17), as well as isolates previously uncultured bacteria from the gut and soil (18, 19). Experiments of even higher throughput are possible by compartmentalizing microbes in droplets of media that are tens to hundreds of micrometers in diameter and separated by immiscible oils and engineered surfactants (20, 21). Because droplets are not limited by the need to microfabricate physical wells or channels, millions of distinct culture volumes can be created in minutes. So far, droplet techniques have been used to isolate uncultured microbes from seawater, soil, and gut communities (18, 22–24); assess microbial cross-feeding (25); track population dynamics of individual bacteria (26); and examine antibiotic sensitivity and commensal-pathogen interactions of human gut and oral microbiota (27, 28). Still, existing droplet microfluidic approaches for assaying bacteria have required combining complex emulsion techniques (water-oil-water) with the use of flow cytometers or custom on-chip droplet sorting devices. These protocol requirements limit the accessibility of droplet technologies for bacterial assays.

Here, we developed a platform to separate, culture, and assay bacteria from human gut microbiota in droplets (MicDrop) using accessible techniques and equipment. A key challenge our method addresses is that of measuring the growth of isolates within distinct microfluidic droplets. To accomplish this, we rely on 16S rRNA genes as intrinsic DNA barcodes that are shared between droplets carrying the same bacterial taxa. This approach in turn allows us to measure growth of taxa in droplets without the need for the use of double-emulsion techniques or droplet sorting. Instead, we combine single-emulsion (water-in-oil) microfluidic droplet protocols with molecular techniques (quantitative PCR [qPCR] and 16S rRNA gene sequencing). These simplified protocols allow us to employ off-the-shelf microfluidic pumps and chips, which are compact enough to fit within typical anaerobic chambers.

To demonstrate the utility of MicDrop for measuring functional differences between human microbiota samples at the level of individual bacterial taxa, we applied the platform to address an outstanding question in gut microbiology research: do individuals differ in the number of gut bacterial species that can degrade complex dietary carbohydrates? Using MicDrop, we characterized dietary polysaccharide metabolism among hundreds of gut bacterial species from nine different people. We found that all individuals harbored microbes that could degrade the carbohydrates examined, and yet the levels of carbohydrate-degrading bacteria differed between the individuals. Gut bacterial taxa could also be broadly categorized by whether they grew on single or multiple polysaccharides. Together, these data suggest rational approaches for prebiotic design and demonstrate the potential of microfluidic droplet assays for comparisons of the growth rates and functions of individual bacterial strains across complex microbial communities.

## RESULTS

### MicDrop: a platform for culturing human gut microbiota in droplets.

To isolate and culture individual gut bacteria from human gut microbiota, we merged concepts from prior microfluidic droplet protocols with high-throughput DNA sequencing (see Materials and Methods). Our protocol first randomly encapsulates individual bacterial cells from gut microbiota into picoliter-sized droplets. Gut microbiota samples are diluted before encapsulation using the Poisson distribution at a loading concentration that optimizes the number of droplets loaded with cells (∼10% to 26%) against the number of droplets loaded with more than one microbe (∼95% to 86% of loaded droplets contain single cells) (see Fig. S1A in the supplemental material) (29). Since many gut bacteria are obligate anaerobes, encapsulation takes place in an anaerobic chamber and droplets are subsequently incubated under anaerobic conditions (Fig. S1B and C). To track bacterial growth, we can avoid having to identify and sort bacteria by assuming that droplets are either empty or loaded with clonal isolates whose progeny share the same 16S ribosomal RNA (rRNA) gene sequence, meaning that genomic material accumulating across all droplets reflects the growth of bacteria grown in isolation. We therefore track isolate growth in droplets at a given time point using bulk bacterial DNA extraction without droplet sorting, followed by DNA sequencing and total quantification (qPCR) of the 16S rRNA gene. To obtain as much taxonomic resolution as possible, we group 16S rRNA sequences into sequence variants (SVs) (30). Unlike the conventional operational taxonomic units (OTUs) that cluster sequences using set dissimilarity thresholds, SVs are constructed using sequencing error models and can typically resolve genetic variation more accurately than OTUs (30). The product of values representing relative SV levels from 16S rRNA gene sequencing and total 16S rRNA gene levels yields an estimate of the absolute levels of each SV across all droplets at the time of sampling.

10.1128/mSystems.00864-19.1 FIG S1 (A) Mathematical model of Poisson distributions at different loading concentrations. Trade-offs exist between the number of droplets loaded with bacteria and how many of those loaded droplets are clonal. MicDrop protocols here balance these trade-offs by loading droplets at means ranging from 0.1 to 0.3 (highlighted region). (B and C) Droplet production in an anaerobic chamber (B) and schematic of experimental setup (C). Cells are encapsulated in droplets, which are formed by flowing the aqueous bacterial suspension through immiscible oil via a T-junction on a microfluidic chip (center). Flow is controlled by two syringe pumps (left). Droplet production may be monitored by a microscope equipped with an LCD display (center). After droplets are generated, they are incubated anaerobically (right) until destructive sampling. (D and E) Droplet culture stability over a 5-day period. Results of bright-field microscopy of cell-seeded droplets from the same sample at 0 (D) and 5 (E) days of culture are shown. Scale bars are 100 μm. (F to K) Single-particle encapsulation and determination of Poisson loading. (Top row) Bright-field microscopy of agar droplets containing a single fluorescent bead 1 μm in diameter. Ultralow-melt agar droplets were produced with the same microfluidic droplet generation protocol used for bacterial cultivation with supplemental heating to 42°C to prevent gelation on chip during droplet production. (Bottom row) Complementary fluorescent microscopy of bright-field images at left, showing a single fluorescent particle per droplet. All scale bars are 40 μm in length. Download FIG S1, TIF file, 2.8 MB. Copyright © 2020 Villa et al. 2020 Villa et al. This content is distributed under the terms of the Creative Commons Attribution 4.0 International license.

**FIG 1:**
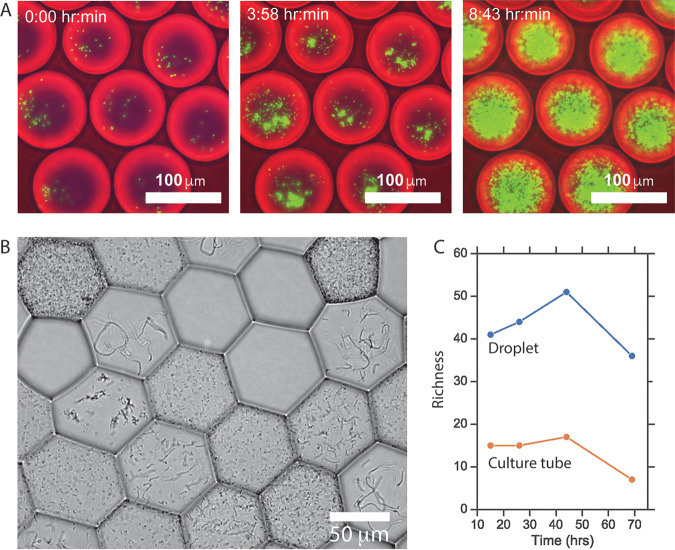
(A) Fluorescently labeled E. coli growing in droplets from h 0 to h 9. We facilitated imaging by overloading E. coli (i.e., most droplets were initially loaded with more than one E. coli cell). (B) Distinct colony morphologies across droplets of an artificial community of five facultative gut anaerobes: Streptococcus agalactiae, Staphylococcus haemolyticus, Enterococcus faecalis, Enterobacter cloacae, and E. coli. Droplets appear hexagonal due to oil evaporation used to flatten the field of view for imaging. (C) Richness of microbial communities isolated and cultured in droplets compared with communities grown without separation in standard bulk culture. Differences in richness were not associated with DNA sequencing depth of samples.

To explore the feasibility of the MicDrop platform, we initially examined bacterial replication and separation in droplets. We observed that aerobic monocultures of fluorescent Escherichia coli were able replicate in droplets (Fig. 1A). Droplet stability experiments suggested that bacteria could be studied in droplets for at least 5 days (Fig. S1D and E). Microscopy showed droplets could be loaded with single particles (Fig. S1F to K). Imaging also provided evidence that we could segregate clonal isolate populations with distinct morphologies and motilities from mixed microbial communities (Fig. 1B; see also Video S1 in the supplemental material). Droplet cultures grown with modified Gifu anaerobic medium (mGAM) were reproducible (within donor replicates, ρ = 0.63 to 0.96, *P* < 0.0001, Spearman correlation), and we found the level of richness of replicate droplet cultures within donors to be lower than the difference in richness between donors (for donor A, median richness = 21, median absolute deviation = 5.3; for donor B, median richness = 62, median absolute deviation = 2.4). Moreover, human fecal microbiota isolated and cultured in droplets exhibited on average 2.8 times greater richness over time than those grown under mixed conditions (Fig. 1C). This finding is consistent with the hypothesis that droplet isolation enables slow-growing microbes to be sheltered from competition with fast-growing bacteria (18, 24, 31).

10.1128/mSystems.00864-19.10 VIDEO S1 Motility of bacteria in aqueous droplets in oil. To enhance imaging and flatten droplets within the focal plane, the oil layer separating aqueous droplets was allowed to partially evaporate. This evaporation leads to droplets taking on a hexagonal shape. Download Video S1, AVI file, 10.1 MB. Copyright © 2020 Villa et al. 2020 Villa et al. This content is distributed under the terms of the Creative Commons Attribution 4.0 International license.

**FIG 2:**
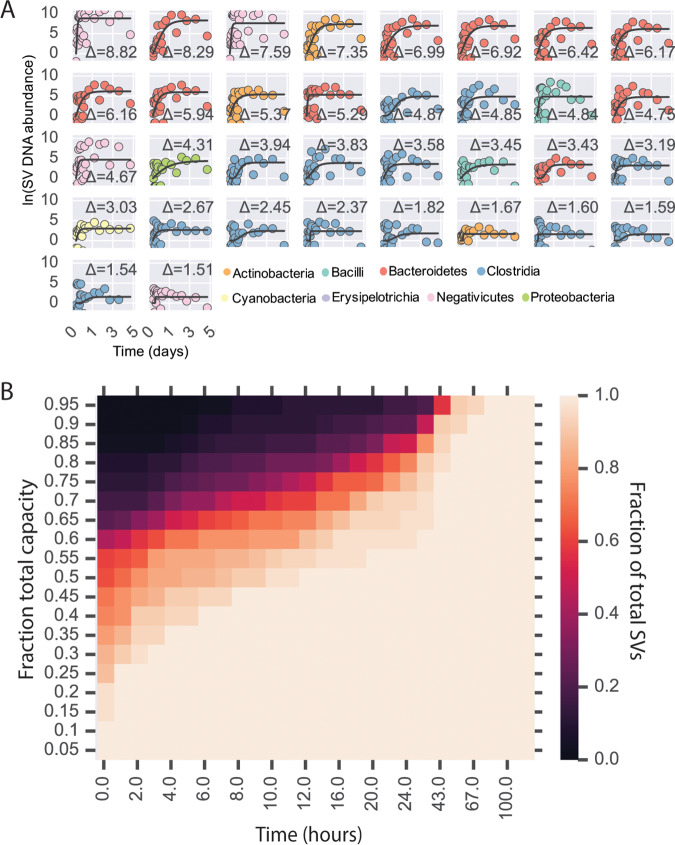
SV growth kinetics in microfluidic droplets. (A) Abundance over time of SVs in MicDrop from a fresh human fecal sample. Levels of growth in replicate droplets were measured hourly for 24 h and daily for the ensuing days. Modified Gompertz growth curves are fitted to a time series (black lines). SVs are colored by taxonomy and sorted according to total growth (curve asymptote height; by an uppercase Greek delta [Δ]), which is denoted on each subplot. Only those SVs inferred to have doubled at least 2.14 times were considered to have been growing and are shown [ln(Δ SV DNA abundance) = ≥1.48; threshold determined using control experiments in Fig. S2]. To ease viewing, curves are shifted vertically such that the *y* intercepts are at the origin. (B) Fraction of SVs that had reached a given fraction of estimated carrying capacity over time. Carrying capacities were inferred from the fitted curves shown in panel A. By 43 h, 97% of SVs had reached 80% of their carrying capacity in droplets.

We then examined the use of DNA sequencing to track bacterial levels in microfluidic droplets. We analyzed whether bacterial DNA levels in MicDrop corresponded to bacterial abundances measured by traditional culture methods. We used MicDrop to culture bacteria from a synthetic mixture of four bacterial strains that were grown under various antibiotic conditions for 24 h. We found that the resulting bacterial DNA levels in droplets corresponded to isolates’ optical densities in reference well plates (accuracy = 89%) (Fig. S2). Next, we examined the timing of harvesting droplets for DNA sequencing when culturing human gut microbiota. We applied MicDrop to a fresh stool sample from a healthy donor. We then created replicate droplet populations and destructively sampled them at hourly intervals for the first 24 h and daily for four subsequent days after inoculation. Among the resulting time series, 94 SVs were detectable in droplets, meaning that they appeared in >5 time points (Table 1; see also Fig. S3). Detected SVs included representatives of the major human gut bacterial phyla (the *Actinobacteria*, *Bacteroidetes*, *Firmicutes*, and *Proteobacteria*) and represented 76% of the inoculating stool sample’s SVs. This fraction is within the range (40% to 78%) noted in previous reports of the fraction of human gut bacterial taxa that can be detected following culture on plates using a single medium (7, 13, 32–34). We further wanted to distinguish within our detected SVs those that were actively growing in our medium from those that were nonviable or nongrowing. We used our antibiotic-based control experiments to establish a limit of detection for growing cells, which we calculated to be a measurement of more than 2.14 cell doublings or a change in ln(SV DNA abundance) of ≥1.48 (Fig. S3). We found that 25% of inoculating SVs exceeded this threshold, meaning that we believed them to be actively dividing in droplets (Fig. 2A). Growing SVs included taxa that were as rare as representing only 0.02% of the starting fecal community. Finally, among the growing SVs, we found that 97% achieved 80% of their final carrying capacity at 43 h after inoculation (Fig. 2B). This observation suggested that sampling microfluidic droplets roughly 2 days after inoculation would provide an informative measure of growth across a diverse set of human gut bacterial taxa.

**TABLE 1:** Number and fraction of microbes from a human stool sample cultured by MicDrop in mGAM^*a*^

Taxonomic level	No. of taxa in inoculum	No. of taxa detected in droplets	No. of taxa in inoculum and detected in droplets	Fraction of inoculum detected in droplets	No. of taxa that grew in droplets	No. of taxa in inoculum that grew in droplets	Fraction of taxa in inoculum that grew in droplets
Phylum	4	5	4	1.00	5	4	1.00
Class	10	11	10	1.00	7	5	0.50
Order	10	14	10	1.00	8	5	0.50
Family	17	21	16	0.94	13	7	0.41
Genus	56	53	40	0.71	20	13	0.23
Sequence variant	89	94	68	0.76	34	22	0.25

a SVs were considered to have been “detected” if present in more than five longitudinal measurements. “Growth” was defined by determination of an inferred number of doublings equal to or greater than 2.14.

10.1128/mSystems.00864-19.2 FIG S2 Results of growth with antibiotics in droplets (A) and 96-well plates (B), using four gut isolates (*Bacteroides* spp. 1, *Bacteroides* spp. 2, Bacteroides thetaiotaomicron, and Enterobacter cloacae) grown in mGAM and six different antibiotic combinations (amoxicillin [100 μg/ml] [amox], amoxicillin plus clavulanate [100 μg/ml] [amoxclav], ampicillin [100 μg/ml] [amp], gentamicin [10 μg/ml] [gent], kanamycin [50 μg/ml] [kan], and ciprofloxacin [5 μg/ml] [cipro]). Growth was measured via qPCR and sequencing (droplets) and OD600 (96-well plates) after 24 h. (C) Receiver operating characteristic (ROC) curve of MicDrop assay results at different growth threshold cutoff values using the data shown in panel B as a reference. A growth cutoff value of doubling of at least 2.14× [Δ ln(SV DNA abundance) ≥ 1.48] maximized the true-positive rate while minimizing the false-positive rate (Youden’s J; maximal value denoted by star on curve) and was used to draw the heat map represented in panel A. Download FIG S2, TIF file, 2.1 MB. Copyright © 2020 Villa et al. 2020 Villa et al. This content is distributed under the terms of the Creative Commons Attribution 4.0 International license.

10.1128/mSystems.00864-19.3 FIG S3 All SVs detected in MicDrop experiment using a human stool sample. Modified Gompertz growth curves are fitted to a time series. SVs are colored by taxonomy and sorted according to total growth (curve asymptote height, indicated by an uppercase Greek delta [Δ]), which is denoted on each subplot. To ease viewing, curves are shifted vertically such that the *y* intercepts are at the origin. Download FIG S3, TIF file, 1.7 MB. Copyright © 2020 Villa et al. 2020 Villa et al. This content is distributed under the terms of the Creative Commons Attribution 4.0 International license.

### A droplet assay for prebiotic consumption by human gut microbes.

To demonstrate how MicDrop could be used to compare the functions of human-associated bacterial communities, we used the platform to measure bacterial utilization of polysaccharides. In typical polysaccharide utilization screens, bacteria are cultured in defined media containing a polysaccharide as the sole carbon source (16, 35). Those microbes that replicate are assumed to be capable of utilizing the polysaccharide and are termed “primary degraders” (36, 37). The biology of primary degraders is of increasing interest because bacterial metabolism of select indigestible polysaccharides (prebiotics) leads to the growth and activity of gut microbes with multiple beneficial impacts on host health (16, 38–42). And yet, bacterial prebiotic metabolism is incompletely understood, in part due to current limitations in bacterial culture-based assays. Human gut microbial culture assays typically do not comprehensively compare the prebiotic consumption potentials of microbiota constituents across individuals; rather, they may investigate only a limited number of type strains or public isolates (15, 16, 43, 44), due to factors such as the cost and availability of reference strains in culture repositories, the effort needed to isolate wild-type prebiotic degraders, and constraints associated with automation of plate handling in anaerobic environments. However, a focus on a select set of prebiotic degraders, particularly without focused comparisons of functional variations in strains across hosts, precludes addressing key issues involving the natural diversity of enteric bacteria, such as (i) the nature of the patterns of prebiotic utilization among the members of the full set of culturable human gut microbiota and (ii) how patterns of microbial prebiotic degradation compare across individuals.

**FIG 3:**
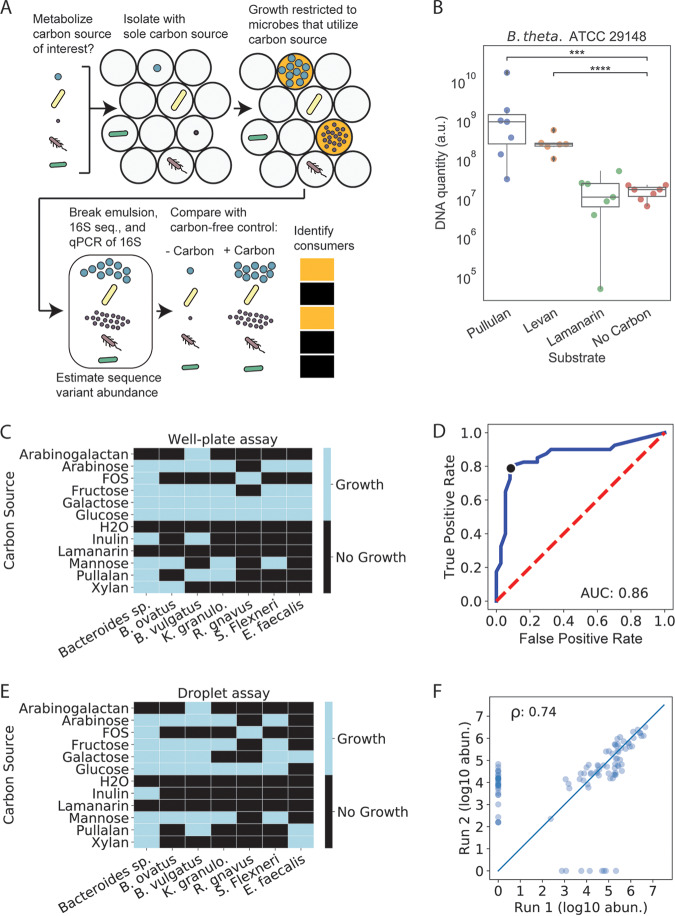
A prebiotic utilization screen based on the MicDrop platform. (A) Schematic of MicDrop prebiotic assay. (B) Droplet monoculture growth of B. thetaiotaomicron in microfluidic droplets measured by qPCR. a.u., arbitrary units. (C) Results of 96-well plate growth of gut bacterial isolates across 11 carbohydrates. FOS, fructooligosaccharide; B. ovatus, Bacteroides ovatus; B. vulgaris, Bacteroides vulgaris; K. granulo., Klebsiella granulomatis; R. gnavus, Ruminococcus gnavus; S. Flexneri, Shigella flexneri; E. faecalis, Enterococcus faecalis. (D and E) Receiver operating characteristic (ROC) curve of MicDrop assay results at different growth threshold cutoff values using data from panel C as a reference. True-positive rate and false-positive rate are defined as true positives/total positives and false positives/total negatives, respectively. (D) The black dot indicates the growth threshold that maximizes the true-positive rate while minimizing the false-positive rate (depicted in panel E). (F) Correlation between two different MicDrop sessions (each carried out in triplicate) on the same frozen fecal sample with five different carbohydrates. Points indicate median growth levels of different SVs across each experimental session.

To investigate whether the MicDrop platform could be used to address these issues of gut microbial prebiotic degradation, we performed a set of validation experiments focused on bacterial prebiotic metabolism (Fig. 3A). Since most human gut microbes previously sampled reached carrying capacity after two days of growth in droplets (Fig. 2B), we carried out each of these validation experiments after 48-h growth periods. First, we loaded a previously characterized type strain, Bacteroides thetaiotaomicron ATCC 29148, into droplets and standard 96-well plates. Consistent with both prior studies (16) and our well plate experiments, B. thetaiotaomicron ATCC 29148 replicated in droplets on pullulan and levan but not on laminarin or a no-carbohydrate control (Fig. 3B). We next tested how the MicDrop prebiotic assay performed using artificial microbial communities assembled from seven human gut isolates (Fig. 3C). Using experiments performed with 96-well plates as our reference (Fig. 3C to E), we found the accuracy, sensitivity, specificity, and false-discovery rate of the MicDrop prebiotic assay to be 87%, 80%, 93%, and 9%, respectively. Finally, to assess the reproducibility of the MicDrop prebiotic assay, we used the same frozen fecal sample from a healthy donor to compare the results of two separate experimental sessions. We observed higher correlation between replicates from the same session (ρ = 0.73 to 0.78, *P* < 0.0001, Spearman correlation) than between replicates across sessions (ρ = 0.57, *P* = 1.67e−17, Spearman correlation). One explanation for the difference in correlations is that our prebiotic assay involved reviving and preparing frozen fecal samples using overnight culture prior to droplet encapsulation and that the microbial communities reassembled in slightly different configurations during the overnight cultures (Fig. S4) (45). Indeed, controlling for microbiota differences between droplet inocula elevated the between-session correlation (ρ = 0.74, *P* = 5.14e−19, Spearman correlation) (Fig. 3F).

10.1128/mSystems.00864-19.4 FIG S4 Comparison between culture inocula from the same frozen stock. (A) Cells were revived from frozen fecal slurries in rich medium (mGAM) overnight to allow cells to recover from freezing. Excess nutrients were then depleted by again culturing bacteria overnight in minimal medium containing glucose and galactose as the sole carbon sources. Following determination of the loading concentration, the bacteria were then washed and diluted prior to encapsulation. (B) Reproducibility of inoculum composition following revival from identical frozen stock sources. Download FIG S4, TIF file, 0.9 MB. Copyright © 2020 Villa et al. 2020 Villa et al. This content is distributed under the terms of the Creative Commons Attribution 4.0 International license.

### Identifying primary degraders from human guts across multiple prebiotics.

**FIG 4:**
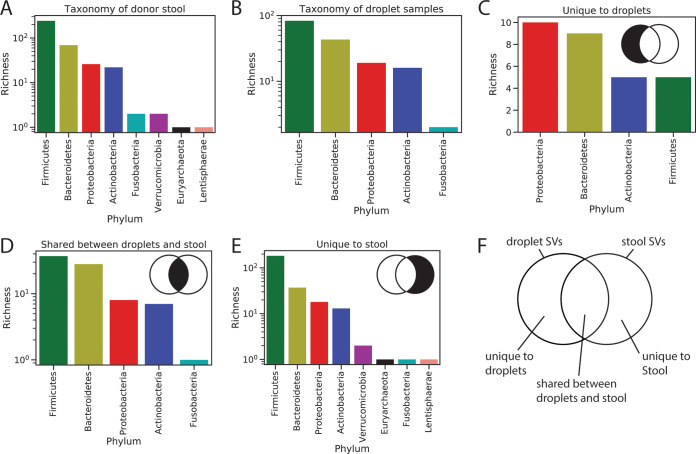
Taxonomic distribution of donor stool and droplet samples used in MicDrop prebiotic assay. (A) Phylum-level counts of taxa found across 9 donor stool samples. (B) Phylum-level counts of taxa that grew in the prebiotic assay. (C to E) Phylum-level counts of SVs unique to droplet growth on the prebiotic assay (C), shared between droplet cultures and donor stool (D), or unique to donor stool samples (E). (F) Venn diagram of overlap of droplet and stool SVs (not scaled by SV number).

Following our validation, we used MicDrop to compare levels of polysaccharide utilization across gut microbiota from nine healthy human stool donors. We assayed growth on three consumer-grade prebiotics (inulin, galacto-oligosaccharides [GOS], and dextrin) and on a laboratory-grade polysaccharide (xylan). Of the 7,092 donor-specific SVs detected in stool by 16S rRNA sequencing, 204 grew in MicDrop on at least one of the screened polysaccharides (primary degraders). An additional 94 SVs grew in droplets that were not detected in corresponding stool samples. These taxa shared between droplet cultures and stool samples included members of major bacterial phyla (*Firmicutes*, *Bacteroidetes*, *Proteobacteria*, *Actinobacteria*, and *Fusobacteria*; Fig. 4). Some of these taxa may have been contaminants; we noticed that 24 taxa found only in prebiotic droplets were also detected in our carbon-free controls. However, droplet-specific taxa might also reflect the elevated sensitivity of culture relative to metagenomics in some scenarios (46, 47). Indeed, we observed that droplet-specific taxa were enriched for members of the *Bacteroidetes* (Fig. 4), which is in line with the primary components of our base assay medium having originally been designed to culture members of this phylum (35). This medium bias may also explain why certain rarer phyla (*Verrucomicrobia*, Euryarchaeota, *Lentisphaerae*) were absent from droplets.

**FIG 5:**
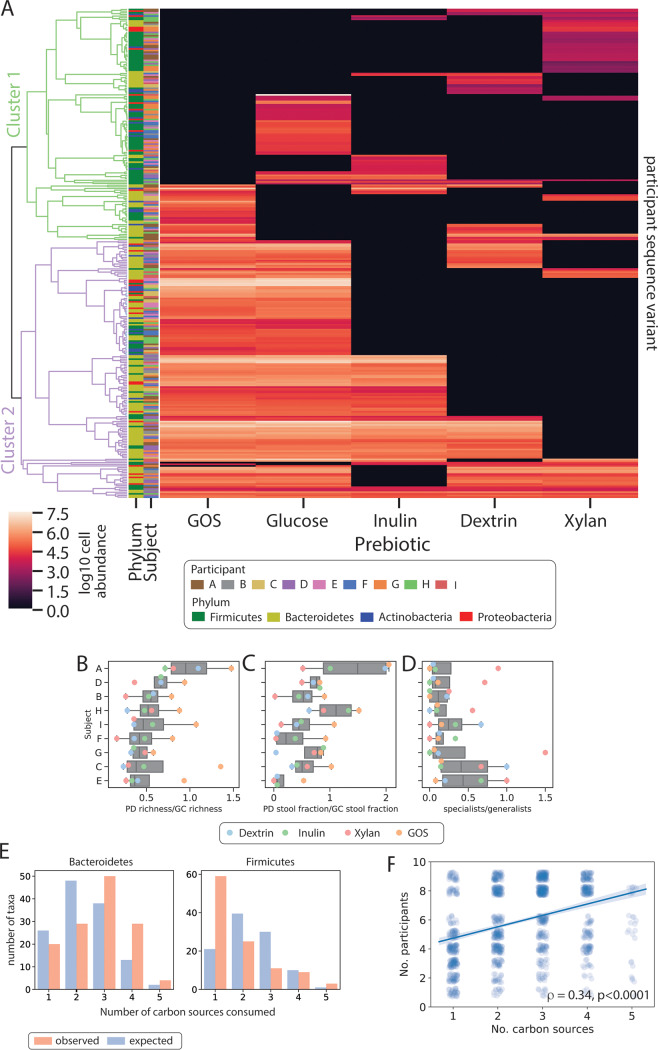
MicDrop prebiotic assay carried out on fecal samples from nine individuals. (A) Microbial carbohydrate preferences for 298 SVs from nine healthy human donors. We defined primary degraders as SVs that grew on at least one polysaccharide. Rows (SVs) were clustered by calculating the Euclidean distance between prebiotic growth profiles. A cutoff between clusters 1 and 2 was set visually to reflect how subtrees of SVs were generally characterized by growth on either a single carbohydrate or multiple carbohydrates. (B to D) The number of primary degraders (B), the stool abundance of primary degraders of a given prebiotic (C), and the ratio of specialists (i.e., those that grew on only one carbon source) to generalists (i.e., those that grew on multiple carbon sources) for each participant and carbon source (D). Primary degraders (PD) were normalized by glucose consumers (GC) to control for potential differences in overall microbiota viability. Participant ordering in panels B to D is sorted by median values of primary degrader counts per participant. (E) Numbers of SVs from a given phylum observed to grow on different numbers of carbon sources (red), and counts expected by chance (blue). (F) SVs plotted according to the number of participants they were found in and the number of carbon sources they degraded. Spearman correlation reported.

We investigated the overall patterns in SV prebiotic utilization observed within the MicDrop dataset. Clustering of prebiotic utilization patterns suggested the presence of two groups of SVs (Fig. 5A). One SV group (cluster 1) was typically characterized by growth on only a single carbohydrate. The other SV group (cluster 2) tended to be able to grow on multiple types of carbohydrates. This clustering pattern motivated us to categorize individual SVs as either “specialists” (cluster 1) or “generalists” (cluster 2) (though we acknowledge that taxa denoted as specialists may grow on other untested carbohydrates). The dichotomy of specialist and generalist SVs may have evolutionary origins. Prebiotic utilization patterns tended to be shared among taxa that were from the same species or SV (*r_M_* > 0.12, *P* < 0.05, Mantel test) but not at higher phylogenetic levels. Such a pattern is consistent with predictions suggesting that closely related taxa would exhibit conserved metabolic traits (48). Bacteria from the phyla *Firmicutes*, *Actinobacteria*, and *Proteobacteria* were also significantly more likely to grow on a single prebiotic than would be expected by chance (*P* < 1e−4, *P* = 0.0011, and *P* = 0.0271, respectively, permutation test), whereas *Bacteroidetes* were more likely to grow on multiple prebiotics (*P* = 0.0012, permutation test) (Fig. 5E). This difference in phylum-level preferences is consistent with previously published estimates that the *Bacteroidetes* harbor 4-fold more glycoside hydrolase and polysaccharide lyase genes on average than *Firmicutes*, *Actinobacteria*, or *Proteobacteria* (36). We observed that the specialist-to-generalist ratio across individuals was significantly less than 1 (median, 0.44, *P* = 3.9e−3, Wilcoxon signed rank test) (Fig. 5D) and that the number of carbon sources that a given SV degraded positively correlated with the number of participants in which the SV was found (ρ = 0.34, *P* = 3.62e−36, Spearman correlation) (Fig. 5F). These observations are consistent with an evolutionary model in which bacteria with more-flexible carbohydrate utilization profiles are more capable of dispersing across and persisting within individuals than SVs with more-restrictive metabolic lifestyles (49).

We then explored the hypothesis that differences in the presence or absence of primary degraders are able to drive interindividual variation in human prebiotic response. Evidence arguing against this hypothesis included the observation that multiple SVs capable of growing on the tested prebiotics were present in all subjects (median, 12.5 ± 6.1) (Fig. 5B). Concordant with human studies showing that even individuals with low prebiotic fermentation *in vivo* exhibit at least some prebiotic fermentative capacity *in vitro* (37), this finding supports the hypothesis that primary degraders are found across most individuals. Still, we also found evidence for interindividual variation in primary degrader composition and abundance. We found that subject identity explained more variation (*R*^2^ = 0.30, *P* = 0.001, permutational multivariate analysis of variance [PERMANOVA]) than prebiotic type (*R*^2^ = 0.16, *P* = 0.001, PERMANOVA) in overall primary degrader growth. We also observed differences in the richness of primary degraders across subjects (*P* = 2.8e−07, two-way ANOVA) and in the relative abundances of primary degraders in inoculating fecal communities (*P* = 3.9e−4, two-way ANOVA) (Fig. 5C). This variation appeared in spite of our having controlled for potential differences in overall microbiota viability due to sample collection and handling, which we performed by normalizing levels of primary degraders to the levels of glucose consumers. Overall, we observed the normalized richness of primary degraders to differ by 2.6-fold across individuals and the normalized stool abundance of primary degraders to differ by 24.7-fold. Thus, while primary degraders are likely present in most individuals, observed differences in polysaccharide metabolism *in vivo* (50, 51) might be due to interindividual variation in primary degrader abundance in the gut.

## DISCUSSION

We have introduced a high-throughput platform that enables functional measurements of individual bacterial taxa from a human microbiota sample (MicDrop). A wide range of human gut bacteria were grown on this platform and exhibited growth kinetics similar to those observed in conventional culture. As a proof of concept, we used MicDrop to perform the most comprehensive characterization of natural variation in dietary polysaccharide utilization among human gut microbiota reported to date. The resulting data suggest that gut bacterial metabolism of prebiotics is carried out by both specialist and generalist bacteria and that the levels of these microbes differ between individuals.

A key benefit of our platform is its level of throughput, which in turn makes it feasible to compare the functions of individual bacterial taxa between multiple microbiota samples. We estimate that, using traditional culture methods, roughly 700 colonies would have needed to have been isolated and genotyped per fecal sample to capture the same level of diversity assayed in our MicDrop prebiotic experiments. While such efforts may be feasible for a limited number of samples, the time needed to process large numbers of samples is prohibitive. In contrast, as sample numbers grow, MicDrop requires only modest increases in effort since additional droplet encapsulation runs require only minutes of extra labor; also, multiplexing techniques enable many droplet cultures to be assayed in a single high-throughput DNA sequencing run. Coupled with our use of compact and off-the-shelf microfluidic pumps and chips, we believe that MicDrop now makes it feasible for researchers to measure how the functions of individual bacterial taxa differ among an array of microbiota samples.

Still, the MicDrop platform has some limitations. In particular, we speculate that some of the error associated with our validation experiments could have been due to the presence of rare multicellular encapsulations during droplet generation. For example, in our prebiotic assays, we might have encapsulated primary degraders with a second nonprebiotic degrading microbe that cross-feeds on the primary degrader’s byproducts of prebiotic degradation. Such coencapsulation would lead to development of a false-positive signal of prebiotic degradation for the second microbe. At our target dilutions, such events are expected to be atypical: an estimated 0.5% to 4% of the droplets consisted of multicellular droplets. Future users of our technology will be able to reduce multiencapsulation by seeding droplets at lower Poisson dilution. And yet, the use of such dilution would represent a trade-off as droplet generation experiments would need to be lengthened in order to maintain the number of droplets containing cells.

These caveats notwithstanding, we were still able to use MicDrop to provide insight into the hypothesis that individuals’ gut microbiota differ in their prebiotic utilization potential (42). Specifically, our measurements suggest that while most individuals harbor gut microbial strains that can utilize common prebiotics, the abundance of these strains may represent a nearly 25-fold range between people. We note that culture-based utilization data from MicDrop could be used to make *a priori* predictions of which individuals in a population would be responders or nonresponders to a prebiotic therapy (52). Such predictions could be used to personalize prebiotic treatments to a given individual (53) or to limit the costly inclusion of individuals who would not respond to treatment in a clinical trial (54). Additionally, our data suggest rational approaches for stimulating the greatest number of gut taxa using the fewest prebiotics. Specifically, combinatorial therapies could be composed of prebiotics that target different groups of gut microbiota. Here, we observed that one of the prebiotics examined (xylan) and the three other prebiotics (inulin, GOS, and dextrin) exhibited relatively little overlap in terms of degrading bacterial species (Fig. 5A; see also Fig. S5 in the supplemental material). Our data predict that a two-prebiotic cocktail involving xylan would be sufficient for stimulating a majority (88%) of the bacterial SVs that grew in our prebiotic assay (Fig. S5).

10.1128/mSystems.00864-19.5 FIG S5 (A) Examination of which prebiotic combinations were observed more and less likely by chance. Plus and minus signs at the bottom of the plot indicate the number of taxa observed for a given combination that were statistically more and less likely to occur at a cutoff value (*P* < 0.05, permutation test), respectively. (B) Fraction of culturable gut microbiota predicted to be stimulated by different combinations of prebiotics based on droplet data. Download FIG S5, TIF file, 1.3 MB. Copyright © 2020 Villa et al. 2020 Villa et al. This content is distributed under the terms of the Creative Commons Attribution 4.0 International license.

Beyond uses in human-associated microbiology, MicDrop could ultimately be applied in environmental microbiology studies. Environmental microbiota often feature levels of diversity similar to or even greater than those of the microbial taxa of human-associated communities, which indicates a similar need for high-throughput bacterial culture (55, 56). Additionally, analysis of microbial phenotypes by culture can reveal important insights into the differences between environmental microbial communities and their habitats. In marine environments, differences in bacterial temperature sensitivity are correlated with seasonal shifts in bacterial ecosystem function (57). In agricultural settings, differing abundances of bacteria exhibiting antibiotic resistance can reflect fertilization strategies (58). And, in grassland habitats, the diversity of bacterial carbon metabolic capabilities can be modulated by the presence of keystone plant species (59). By enabling functional comparisons between individual bacterial from a variety of samples, MicDrop may therefore also provide unique new datasets that will improve our understanding of environmental microbiology (48, 60, 61).

## MATERIALS AND METHODS

### Overall MicDrop procedure.

Droplets were made on a microfluidic chip (6-junction droplet chip; Dolomite Microfluidics). The bacterial media used varied by assay. For the oil phase, we used a fluorinated oil and surfactant mixture consisting of 1% Pico-Surf (Sphere Fluidics)–Novec 7500 (3M). One day prior to initiation of the droplet assay, all reagents, including carrier oil, culture media, and carbon solutions, were equilibrated to the anaerobic atmosphere in an anaerobic chamber (Coy). The fecal inoculum optical density at 600 nm (OD_600_) was recorded, and the inoculum was diluted according to the Poisson distribution using the equation $P\left(n,\bar{n}\right)=\frac{{\bar{n}}^{n}e^{-\bar{n}}}{n!}$, where n is the droplet occupancy (i.e., 0.1 cells/droplet) and $\bar{n}$ is the average number of cells per droplet given by the equation $\bar{n}=\text{ρ}V$, where *V* is droplet volume and ρ is cell density. Assays were performed using $\bar{n}$ values of 0.1 to 0.3 to minimize the number of droplets loaded with more two or more cells (see Fig. S1 in the supplemental material). Thus, for a fixed droplet volume and $\bar{n}$ value, the target cell concentration can be obtained from the equation $\text{ρ}=K\frac{\bar{n}}{V}$, where *K* is a constant that converts CFU counts per milliliter to optical density at 600 nm (OD_600_) determined from replicate CFU assays on blood agar plates (catalog no. A10, Hardy). We note that in the growth curve experiments, loading concentrations were chosen by loading droplets at different dilutions (*K*) and then counting empty droplets to calculate the fraction of droplets containing clonal populations of bacteria (Fig. S1) as previously described (13). A loading dilution was then chosen such that as close to 20% of droplets as possible were clonally loaded. This dilution corresponds to a proportion of droplets with multiple bacteria of <3%. We estimate that at our loading concentrations and working volumes, we typically generated on the order of a million droplets loaded with a single microbe per experiment. Increasing the number of generated droplets by a factor of up to 3 did not increase the richness of the droplet cultures (data not shown). Syringe pumps were used to control the flow rates of the oil and cell suspension (NE-1000 single-syringe pump; New Era Pump Systems). Following the culture period, droplets were loaded into chambered slides (catalog no. C10283, Invitrogen) or directly onto glass slides and observed with phase-contrast and/or dark-field microscopy (Nikon) to examine growth and the appropriate loading level. All steps of cell encapsulation and culture were performed in an anaerobic chamber.

### Collection and preparation of fecal inocula.

Stool was collected from human donors under a protocol approved by the Duke Health Institutional Review Board (Duke Health Institutional Review Board [IRB] Pro00049498). Inclusion criteria limited donors to healthy subjects who could provide fecal samples at no risk to themselves, had no acute enteric illness (e.g., diarrhea), and had not taken antibiotics in the previous month. Stool samples were collected in a disposable commode specimen container (Fisher Scientific, Hampton, NH). Intact stool was moved within roughly 15 min of bowel movement to anaerobic conditions. The sample was prepared for inoculation in an anaerobic chamber (Coy). A 5-g stool aliquot was weighed, placed into a 7-oz filtration bag (Nasco Whirl-Pak), and combined with 50 ml of mGAM (Gifu anaerobic medium [HiMedia], with the addition of 5 mg/liter vitamin K and 10 mg/liter hemin [32]) that was prereduced overnight in an anaerobic chamber. The mixture was homogenized in a stomacher (Seward Stomacher 80) for 1 min using the normal-speed setting under atmospheric conditions to make a total of 100 ml of inoculum. The supernatant was filtered through a 50-μm-pore-size CellTrics filter, diluted, and loaded into droplets. A limited number of human stool samples were collected under another protocol approved by the Duke Health Institutional Review Board (Duke Health IRB Pro00093322). These samples were used to examine the within-subject reproducibility of droplet cultures and the effect of increasing the number of droplets on droplet culture diversity. Participant enrollment for this protocol was limited to healthy individuals between the ages of 18 and 70 years. Exclusion criteria included food allergies to milk or wheat/gluten, a history of irritable bowel syndrome, inflammatory bowel disease, type 2 diabetes, chronic kidney disease or reduced kidney function, intestinal obstruction, untreated colorectal cancer, antibiotic treatment within the previous 1 month, pregnancy, and breastfeeding. Participants were asked to self-collect stool using provided sampling kits. Sampling kits consisted of an adhesive waxed paper toilet accessory (catalog no. OM-AC1; DNA Genotek, Ottawa, Ontario, Canada) and a fecal specimen collection tube (catalog no. 109120; Globe Scientific, Mahwah, NJ, USA). Home-collected stool samples were stored in personal freezers and were then brought to the laboratory on a weekly basis by participants. Participants were also provided with an insulated bag and an ice pack to enable cold transport of samples from home freezers to the laboratory, where the samples were placed in a locked −20°C drop-off freezer. This −20°C drop-off freezer was emptied on a weekly basis, and samples were transferred to a −80°C freezer to be stored until use. Aliquots were made from homogenized stool in mGAM using a stomacher and filtration bag as described above. Then, stool aliquots were mixed 50:50 with a 50% solution of glycerol–phosphate-buffered saline (PBS) and frozen at −80°C for later use.

### Droplet DNA extraction, PCR amplification, and DNA sequencing.

To extract DNA from droplets, excess oil was removed by pipetting and water-in-oil emulsions were broken by adding an equal amount of 1H,1H,2H,2H-perfluoro-1-octanol (PFO; VWR) and were subjected to a brief period of vortex mixing. Then, the samples were briefly centrifuged (<200 × *g*) to separate the aqueous and oil phases by density. The aqueous solution was transferred to a new tube, and DNA was extracted using an UltraClean kit (Qiagen catalog no. 12224). DNA was extracted from stool samples in our time-series experiments using a 96-well PowerSoil kit (Qiagen catalog no. 12888). For all samples, the V4 region of the 16S rRNA gene was barcoded and amplified from extracted DNA using custom barcoded primers and previously published protocols (62, 63). 16S rRNA amplicon sequencing was performed on an Illumina MiniSeq sequencing system with paired-end 150-bp reads. We chose to analyze only those samples that had more than 5,000 reads to remove outlying samples that might have been subject to the accumulation of library preparation or sequencing artifacts. Sample read depth data are provided in Table S1 in the supplemental material. Total abundances of bacteria from droplet cultures were estimated by qPCR for bacterial 16S rRNA using the primers used in the DNA sequencing protocol. Amplification during the qPCR process was measured with a real-time PCR system (CFX96 real-time system; BioRad) using E. coli DNA at a known cell concentration as a reference.

10.1128/mSystems.00864-19.7 TABLE S1 16S rRNA sequencing sample metadata and read depth. Download Table S1, XLSX file, 0.02 MB. Copyright © 2020 Villa et al. 2020 Villa et al. This content is distributed under the terms of the Creative Commons Attribution 4.0 International license.

### Identifying sequence variants and taxonomy assignment.

DADA2 was used to identify SVs (30). Custom scripts were used to prepare data for denoising with DADA2 as previously described (64). Reads were then demultiplexed using scripts in Qiime v1.9 (65). SVs were inferred by DADA2 using error profiles learned from a random subset of 40 samples from each sequencing run. Bimeras were removed using the function removeBimeraDenovo with tableMethod set to “consensus.” Taxonomy was assigned to sequence variants using a naive Bayes classifier (66) trained using version 123 of the SILVA database (67). For examination of the growth dynamics of the human gut microbiota, only forward sequencing reads were analyzed. Downstream analysis of sequence variant tables was performed using R (ver. 3.4.2) and Python (ver. 2.7.6). PERMANOVA was run in R using adonis in the vegan package (ver. 2.5-2).

### Growth dynamics of human gut microbiota.

To estimate SV growth curves using MicDrop, we collected a total of 70 separate microfluidic droplet aliquots for destructive longitudinal sampling. Droplets were generated according to the MicDrop protocol described above. We used modified Gifu anaerobic medium (mGAM) (HiMedia) in our droplets, with the addition of 5 mg/liter vitamin K and 10 mg/liter hemin. Each aliquot of 200 μl of droplets was incubated at 37°C in an anaerobic chamber. Aliquots were destructively sampled in triplicate, hourly, from h 0 to h 24 after droplet making and in duplicate once a day from h 24 to h 127 after droplet making.

Growth curves were fitted using a combination of 16S rRNA qPCR and DNA sequencing data. To minimize the potential for poorly fitted growth curves, SVs were required to have been detected by DNA sequencing in >5 samples to be included in curve fitting. To avoid numerical instabilities associated with taking the log or dividing by zero, a pseudocount value of 1 was added to the sequence variant count table prior to normalization to relative abundances. Relative abundances of each SV were then determined by dividing the number of counts associated with each SV in each sample by the total read counts in the sample. The concentration of each taxon was then estimated by multiplying the relative abundances of SVs by the 16S rRNA concentrations determined by qPCR. Technical replicates constituted distinct data points in these calculations. We used the SciPy Python package (v0.19.1) to fit a modified Gompertz equation (68) to which we added an additional term to the resulting dataset to account for differences in starting abundances using the equation $y=\mathrm{Aexp}\left\{-\exp\left[\frac{\text{μ}\cdot e}{A}\left(\text{λ}-t\right)+1\right]\right\}+A_{0}$, where μ is growth rate, *A* is carrying capacity, λ is lag time (or the time it takes for a bacteria to reach logarithmic growth), and *A*_0_ accounts for the relative abundances of the different SVs in the inoculum. We fitted curves using the module scipy.optimize.least_squares with the robust loss function “soft_l1.” Parameter bounds were also used to minimize the optimization search space. We set lower bounds of *A* = 0, λ = −50, μ = 0, and *A*_0_ = 0 and upper bounds of *A* = 15, λ = 12, μ = 2.6, and *A*_0_ = 15. We selected bounds by considering both biological feasibility and parameter sensitivity analyses (Fig. S6). Our upper bound for growth rate (μ = 2.6) represented a doubling time of 15 min, which we based on the highest growth rates observed in an anaerobic bacterium (69). The upper bounds on *A* and *A_0_* were set to the maximum amount of DNA measured across replicate MicDrop samples from the human fecal inoculum. The upper bound for λ, which represents the lag time until the exponential-growth stage is reached (70), was set at 12. The assigning of a lower bound of 0 for *A*, *A_0_*, and μ reflected our choice not to model negative growth. A lower bound for λ was selected by sensitivity analysis (Fig. S6), which revealed that the choice of a bound of 0 led to fitted λ values regularly collapsing to our boundary limits. We also found that fitted curves were sensitive to starting parameters. To ensure a broad search of parameter space, we initialized each curve fit multiple times (*n* = 100) with starting values randomly distributed between the bounds of each parameter. Fitted growth rates often collapsed to the maximum μ value tolerated; we therefore retained only those fits where the growth rates were at least slightly below our upper bound for μ (μ < 2.5). Among the remaining fitted curves, we analyzed the one with the lowest loss-of-function value. In our analyses of SV growth in human fecal samples, we defined total SV levels as *y*(127 h) − *y*(0 h).

10.1128/mSystems.00864-19.6 FIG S6 Sensitivity of curve fitting to parameter bounds. *x*-axis data depict each fit parameter as defined by bounds described in Materials and Methods. *y*-axis data reflect fit parameters with alternative parameter bounds. Each point represents a fit parameters for distinct SV. Download FIG S6, TIF file, 1.9 MB. Copyright © 2020 Villa et al. 2020 Villa et al. This content is distributed under the terms of the Creative Commons Attribution 4.0 International license.

### MicDrop prebiotic assays using human stool samples.

Stool aliquots from the samples collected under the IRB protocol described in the “Collection and preparation of fecal inoculum” section were used for MicDrop growth dynamics assays. Aliquots were drawn from nine healthy donors (7 men, 2 women) between the ages of 35 and 53 years. To facilitate the carrying out of prebiotic assays simultaneously across a range of donors, we used frozen gut microbiota in these experiments. Fecal slurries were made at 10% (wt/vol) using mGAM and a stomacher (Seward) that homogenized fecal samples for 1 min. Then, slurries were mixed 50:50 with 50% glycerol and stored at −80°C for later use. Cells were revived from frozen stock in rich medium (mGAM; see the “Growth dynamics of human gut microbiota” section) for 18 h to allow cells to recover from freezing. Bacteria were then cultured in minimal medium (Table S2) containing glucose and galactose (Sigma) as the sole carbon sources to deplete excess nutrients (71). Following determination of the loading concentration, the bacteria were washed twice by centrifugation (2 min at 14,000 × *g*) to remove free monosaccharides and resuspended in 2× minimal medium without a carbon source. Bacteria were filtered using a 50-μm-pore-size filter (CellTrics; Sysmex) to remove multicell clumps. The filtered microbiota suspension was then added to prebiotics in a 50:50 mixture of 1% prebiotic solution and 2× minimal medium (Table S2). To prevent chip fouling during droplet generation, the oil inlet was equipped with 10-μm-pore-size inline filters (P-276; IDEX). Droplet generation in the anaerobic chamber was monitored using a bright-field microscope (Celestron). Droplet cultures were stored in 5-ml polypropylene tubes (Falcon) with the caps closed in an anaerobic incubator at 37°C. Following the second day of incubation, cultures were moved to a −20°C freezer for storage prior to DNA extraction. DNA was extracted from all stool and microfluidics samples in prebiotic experiments using UltraClean kits (Qiagen catalog no. 12224).

10.1128/mSystems.00864-19.8 TABLE S2 Table of medium formulation and carbon sources used for the prebiotic assay. Download Table S2, XLSX file, 0.01 MB. Copyright © 2020 Villa et al. 2020 Villa et al. This content is distributed under the terms of the Creative Commons Attribution 4.0 International license.

### Validation of prebiotic utilization assays.

To validate the MicDrop prebiotic assay, we generated reference data on carbohydrate preferences using an artificial community of seven wild-type gut isolates from our culture collection (Table S3). These isolates were collected from healthy stool donors under Duke Health IRB Pro00049498. Isolates were obtained by combining 5 g of stool with 25 ml of mGAM culture media followed a brief period of vortex mixing. Following centrifugation at 175 × *g* for 10 min, the supernatant was used to prepare streak plates on 5% sheep blood agar (catalog no. A10, Hardy) and cultivated for 4 days. Colonies were picked and restreaked on individual plates three times and transferred to liquid culture in mGAM to prepare a frozen stock mixed in 50% glycerol. Fecal isolates were assigned taxonomy by sequencing the 16S rRNA gene (primers 8-27F [AGAGTTTGATCCTGGCTCAG] and 1512-1429R [ACGGYTACCTTGTTACGACTT]).

10.1128/mSystems.00864-19.9 TABLE S3 List of bacterial strains used in this study. Download Table S3, XLSX file, 0.01 MB. Copyright © 2020 Villa et al. 2020 Villa et al. This content is distributed under the terms of the Creative Commons Attribution 4.0 International license.

Fecal isolates were grown in both 96-well plates and the MicDrop prebiotic assay described in the preceding paragraph. Following the procedure described in the “MicDrop prebiotic assays using human stool samples” section, well plates were prepared with minimal medium (Table S2) and a carbohydrate as a sole carbon source (Table S2). A 10-μl aliquot of bacterial suspension was added to 200 μl of medium in 96-well plates and incubated in a humidified container for 2 days at 37°C. All culture experiments were performed in an anaerobic chamber. Following the culture period, the optical density at 600 nm of each well was examined using a plate reader (CLARIOstar; BMG Labtech). Following published protocols (16), isolate growth in plates was normalized to the maximum growth rate for each microbe. To classify isolates as either “growing” or “not growing,” a threshold of 20% of maximum growth was applied to the plate data; growth at levels above 20% was considered representative of growth on the carbon source of interest. The isolates used in the well plate analyses were then mixed evenly into an artificial community and examined using the MicDrop prebiotic assay described above. MicDrop experiments were performed in triplicate. Growth thresholds for the MicDrop assay were determined by first assigning preprocessing sample qPCR values a value of 0 if they indicated overall growth below the mean levels seen with no-carbon controls. Then, relative SV abundance data were converted to absolute SV abundances by multiplying each sample by the corresponding qPCR value. Median SV abundances were then calculated across replicates, and values representing SV abundances from matched no-carbon controls were subtracted from each sample. An optimal SV growth threshold for determining growth on a carbohydrate in MicDrop was obtained by applying Youden’s J index across all possible threshold values, with the well plate data as the reference. A growth threshold of 88% maximized this index and was used in subsequent experiments on fecal samples.

### Analysis of the proportions of generalist taxa and specialist taxa by phylum.

Specialists were defined as taxa that grew on only one carbon source, whereas generalists were defined as those that grew on two or more carbon sources. To determine if the number of generalists or specialists was greater than that expected by chance, the droplet carbon consumption table was randomly shuffled to generate a permuted distribution. Then, the fraction of 10,000 permuted distributions in which the number of generalists or specialists was above the observed value was calculated for each phylum and lifestyle combination (e.g., *Actinobacteria*-generalist, *Actinobacteria*-specialist, *Bacteroidetes*-generalist, and so on). All phylum and lifestyle comparisons in which fewer than 5% of the permuted runs were above the observed value for generalists/specialists were reported. Relationships between similarity of carbohydrate utilization patterns of taxa and phylogenetic distance were calculated using the Mantel test. The test was carried out at different phylogenetic levels using the phyloseq R package.

### Data availability.

The 16S rRNA nucleotide sequences generated in this study have been made available at the European Nucleotide Archive under study accession number PRJEB33065.
