# Supplementary figures and images for: Interindividual Variation in Dietary Carbohydrate Metabolism by Gut Bacteria Revealed with Droplet Microfluidic Culture
Source: mSystems. 2020 Jun 30;5(3):e00864-19. doi: 10.1128/mSystems.00864-19 (PMC7329328; doi:10.1128/mSystems.00864-19)

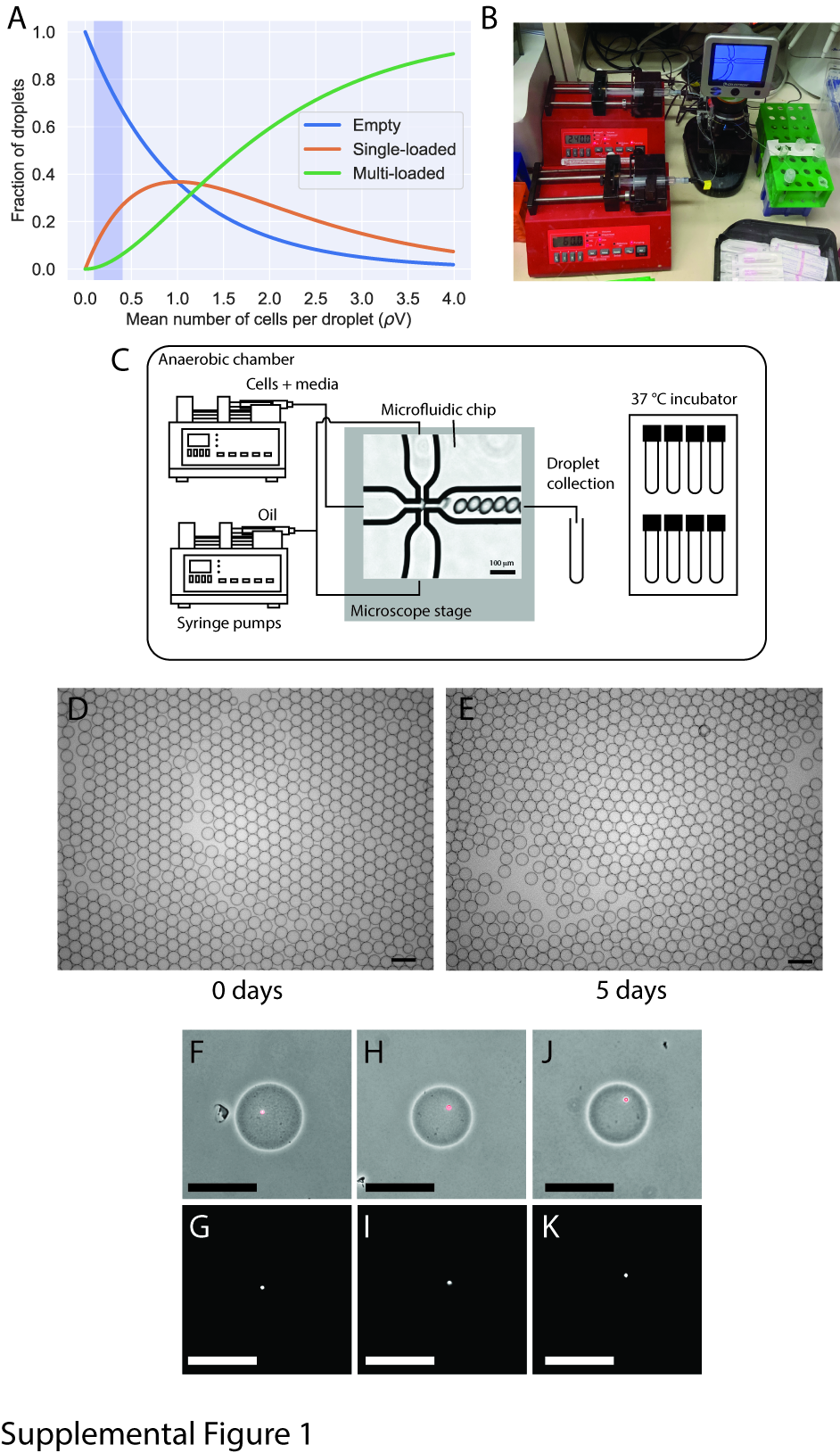

Supplement: FIG S1 [file mSystems.00864-19-sf001.tif]

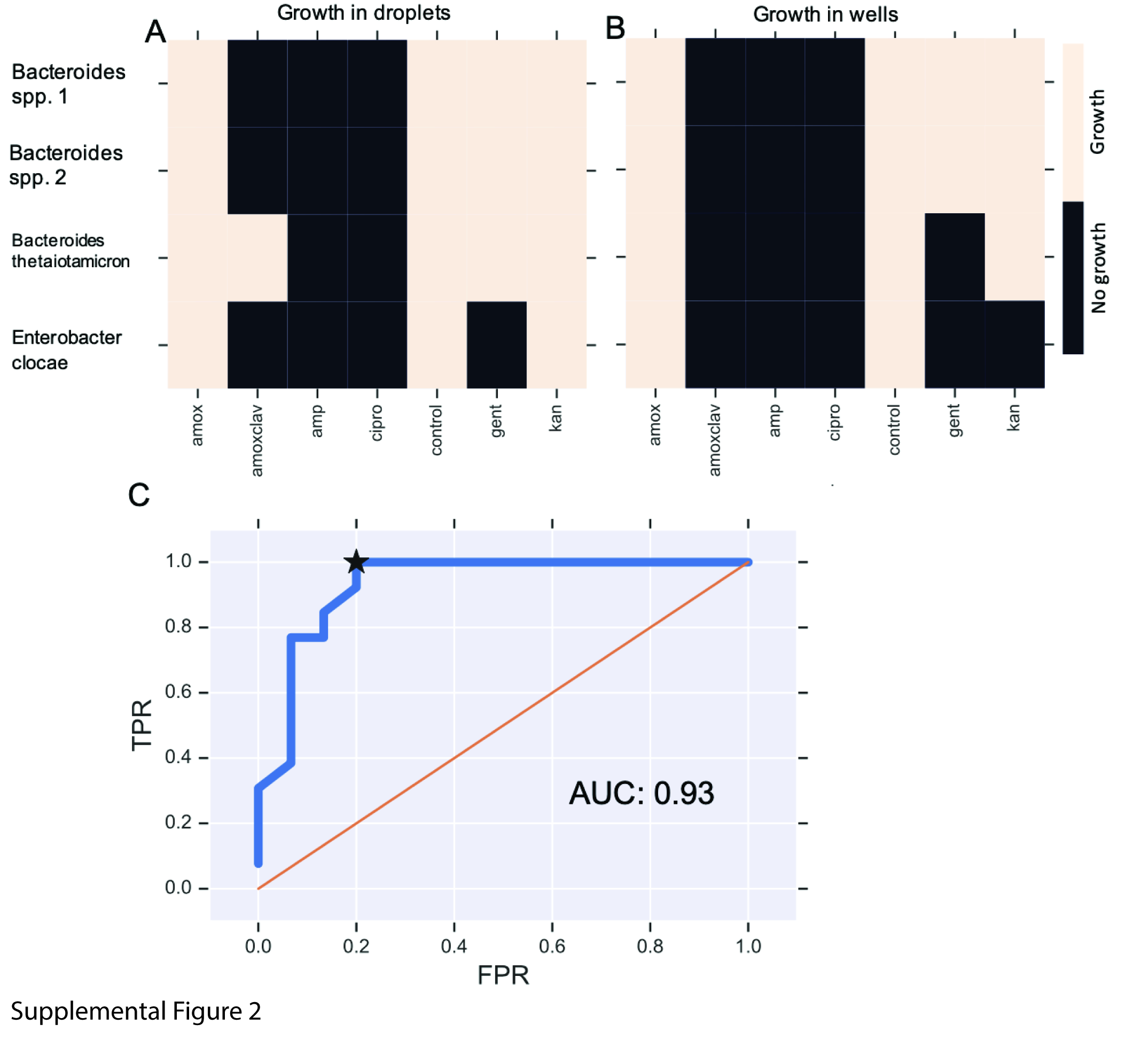

Supplement: FIG S2 [file mSystems.00864-19-sf002.tif]

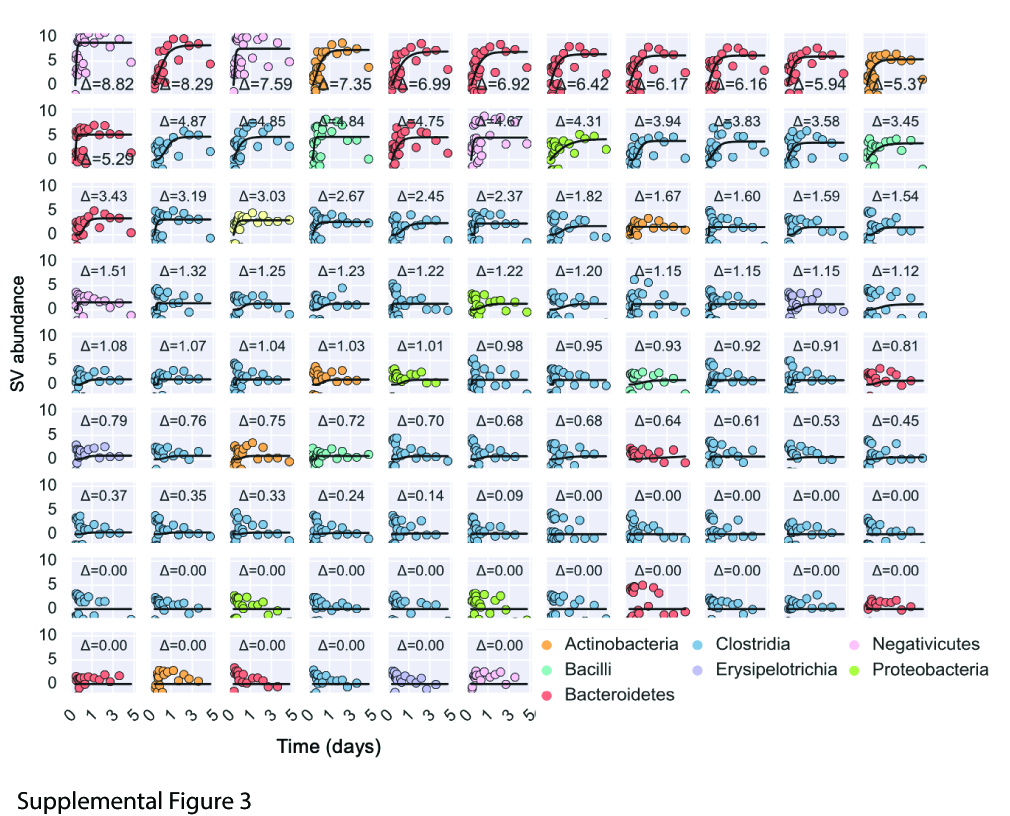

Supplement: FIG S3 [file mSystems.00864-19-sf003.tif]

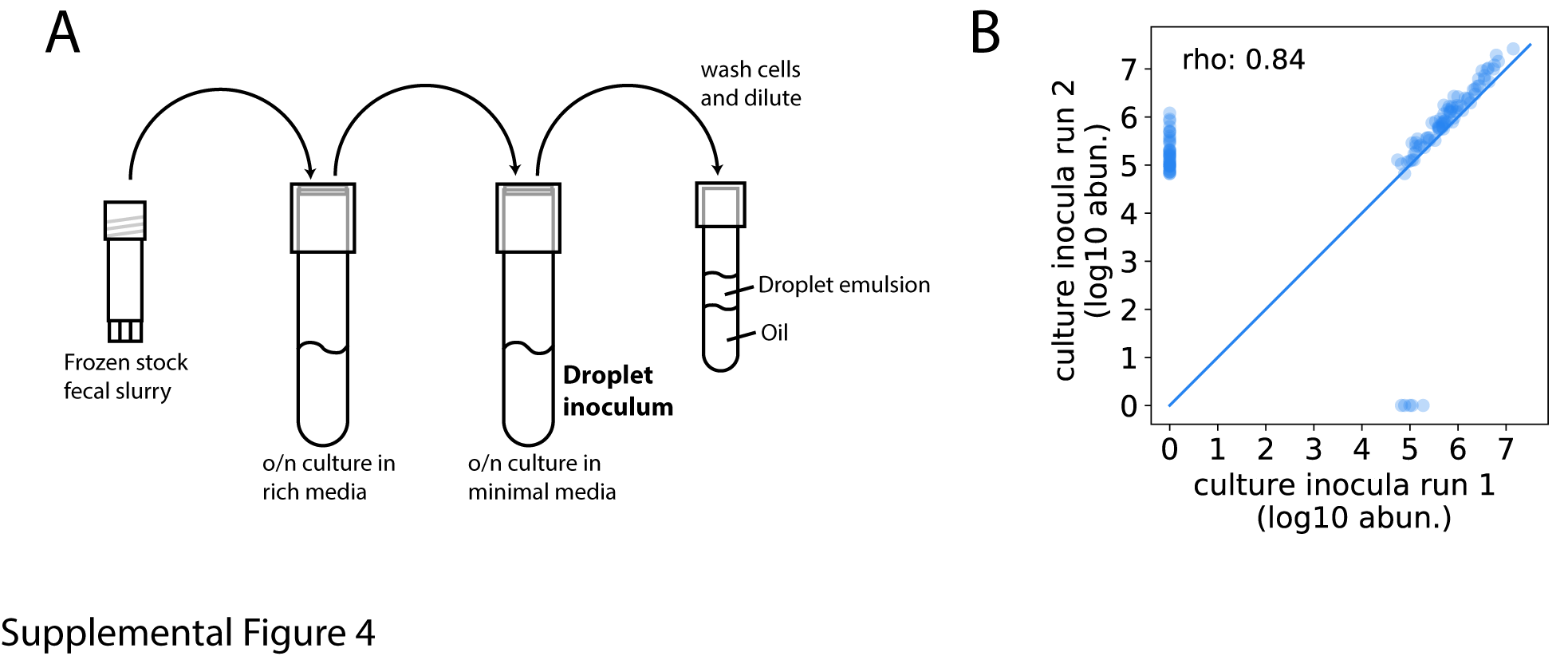

Supplement: FIG S4 [file mSystems.00864-19-sf004.tif]

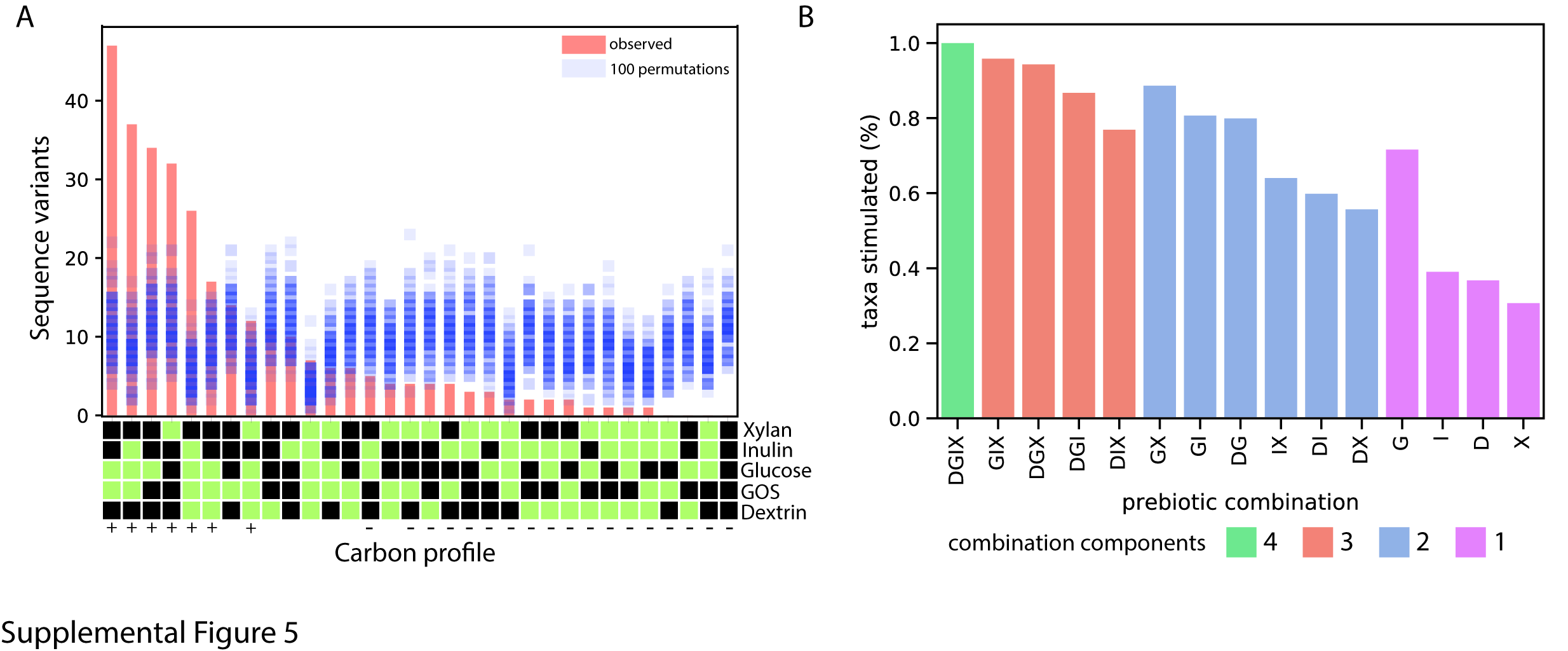

Supplement: FIG S5 [file mSystems.00864-19-sf005.tif]

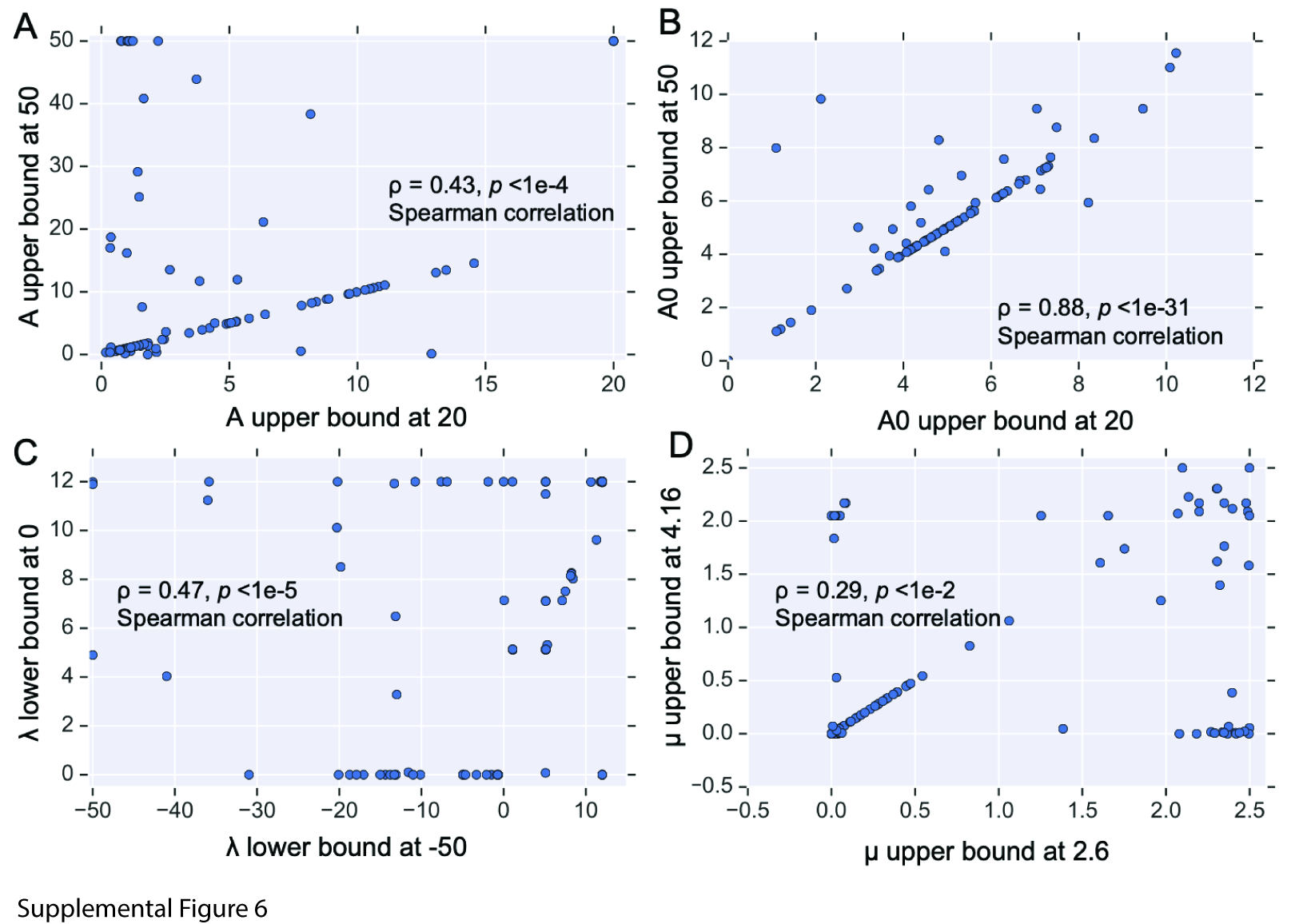

Supplement: FIG S6 [file mSystems.00864-19-sf006.tif]
